# Application of a “Staggered Walk” Algorithm for Generating Large-Scale Morphological Neuronal Networks

**DOI:** 10.1155/2012/876357

**Published:** 2012-09-30

**Authors:** Jack Zito, Heraldo Memelli, Kyle G. Horn, Irene C. Solomon, Larry D. Wittie

**Affiliations:** ^1^Department of Computer Science, Stony Brook University, Stony Brook, NY 11794-4400, USA; ^2^Department of Physiology and Biophysics, Stony Brook University, Stony Brook, NY 11794-8661, USA; ^3^Program in Neuroscience, Stony Brook University, Stony Brook, NY 11794-5230, USA

## Abstract

Large-scale models of neuronal structures are needed to explore emergent properties of mammalian brains. Because these models have trillions of synapses, a major problem in their creation is synapse placement. Here we present a novel method for exploiting consistent fiber orientation in a neural tissue to perform a highly efficient modified plane-sweep algorithm, which identifies all regions of 3D overlaps between dendritic and axonal projection fields. The first step in placing synapses in physiological models is neurite-overlap detection, at large scales a computationally intensive task. We have developed an efficient “Staggered Walk” algorithm that can find all 3D overlaps of neurites where trillions of synapses connect billions of neurons.

## 1. Introduction

Simulating brain structures with large-scale neuronal models lets researchers precisely manipulate features of simulated neural tissues and observe both local and global properties of neural systems. During the last decade, large-scale brain modeling has risen in prominence, with a wide range of publications on brain-scale models[1–3].

Most large-scale modeling research groups focus either on networks that are highly realistic down to the individual axon collaterals and dendrite branches of each neuron [4] or on systems simplified enough to simulate in near real-time on massively parallel hardware [1, 2]. Rather than emphasizing details or simulation speed, our group is more interested in a balanced approach that capitalizes on general structural connectivity and data acquired through multiunit electrode experiments, diffusion tensor imaging, and connectomics studies with stacked slices of brain tissues stained for scanning [4–6]. To develop and test our model-creation code, we have derived parameters for cerebellar models from the detailed connection and density data for the cerebellar cortex in the compendium by Eccles et al. [7].

Large-scale neuronal models range in accuracy from simple, randomly probabilistic networks [8, 9] to realistic neuronal mappings [4]. The level of detail we need for our models is roughly at the tissue level [10], where probabilities of connectivity between distinct volumes of neural tissue and specified neuronal groups can be derived well enough to create alternative models for comparison. The resulting parameters allow for the generation of microcircuitry for particular areas of the brain. The microcircuits can be repeated, with small changes, up to millions of times in some brain regions [10, 11]. 

A critical and complex part of large-scale neuronal modeling is the creation, or initialization, of the myriad of specific details within the model. Even though many modeling studies focus on analyzing and improving simulation run times, the setup and initialization of models with trillions of details can be time intensive and represent significant computational challenges when connectivity patterns are complex [12]. Designing and implementing fast algorithms for model initialization can increase the speed of simulations and make the creation of scalable models more straightforward [12, 13]. 

Synapse placement is of great concern in large-scale models, since synaptic connection patterns control the flow of excitation and inhibition through a neuronal network. A typical approach to initialize synapse placement is to prespecify a few pools of interconnected neurons and define the likelihood that any two neurons in a given pool are connected by a synapse[8]. 

Rather than specifying neuronal pools, our approachdraws from bothstatistical connectivity and spatial location mappings. Instead of defining neuronal pools without any sense of spatial geometry, we placeneurons in a three-dimensional (3D) Cartesian (*XYZ*) space. Each neuron has axonal and dendritic synaptic regions at preset positions relative to its soma. We let each neuron form connections with other neurons within 3D overlap volumes of their synaptic regions. In this way, synapse locations are based on the spatial proximity of axons and dendrites by a method which is more detailed than placing synapses between randomly selected neurons, but less detailed than finding them where axonal and dendritic arborizations touch [4].

The most critical step for our approach is determining axonal-dendritic intersections once all volumes of potential connectivity have been specified by placement of all neuron instances. The task requireswalkingthroughthree-dimensional spaceand determining where synaptic volumes overlap. For neuronal simulations involving many millions of neurons, finding overlapping volumes poses a significant computational challenge. 

To perform neurite-overlap detection efficiently, we have developed a practical algorithm that performs a walk through axonal and dendritic volumes. There are two variants of the algorithm: one “Staggered Walk” (SW) that performs the walk blindly in one predetermined dimension (e.g., *X*) and the other “Staggered Walk Dynamic” (SWD) that uses a quick method to determine the optimal dimension for starting the staggered walk. 

The algorithm is particularly useful in the rapid determination of connectivity between millions of neurons in moderate-scale to large-scale models. Staggered walks provide an automated method to create verifiable simulation models by reproducibly specifying details of neuron placement and synaptic connectivity. Our algorithm scales well and within hours permits the rapid creation, or replication, of models containing trillions of synapses. 

## 2. Methods and Models

Our method uses C++ programs crafted to give the same results on many different computers. Models are specified by textual descriptor files. Results of model creation are checked by visualization programs that show neuronal soma locations and synaptic connections.

### 2.1. Computers and Programming Languages

Our staggered walk algorithm is implemented in C++ and uses the C++ Standard Template Library (STL) for data structures [14]. Our program compiles and runs on multiple computer platforms. It has been tested on a Windows desktop and laptop, a Mac laptop, and a Blue Gene supercomputer. All tests resulted in identical synaptic connectivity regardless of computer platform and degree of parallelism. At first, each C++ compiler generated different random number sequences even from the same initial seed. To ensure cross-platform uniformity of random numbers, we implemented a generator of random numbers based on Marsaglia's method [15]. We also have coded a graphical visualization tool to view the structures built by our algorithm. The visualizer is in C++ using the OpenGL graphics library [16]. 

### 2.2. Model Description Parameter Files

Our approach to building brain models starts with the creation of detailed parameter specifications. The program reads in a parameter text file that statistically describes the neuronal and synaptic configuration of the brain tissues to be modeled. Our program can be modified to accept input files in other formats, such as neuronal description parameters in NeuroML [17].

We have tested our algorithm with cerebellar models based on statistical and morphological data collected from the literature [7, 18, 19] for humans and other mammals. Our initializer reads an input text file that describes the neuronal and synaptic configurations for a structured neuronal model. In Algorithm 1, we present an excerpt from a parameter file that produces human cerebellar models. 

The input parameter file contains a list of cell types, what pairs of cell types can form synapses, the density of each type of soma in the model space, the axonal and dendritic process shapes and positions relative to the soma of each instance of a cell type, plus statistical properties about synapse concentrations and whether they are inhibitory or excitatory. See the Appendix section for more details. The distances of each synapse from its axonal and dendritic somas combined with parameters specifying propagation speeds in axons and speeds at different distances within dendritic trees determine propagation delays (and attenuations) for action potentials passing though each synapse. To lessen computer memory needs and execution times, our present modeling system does not consider branching details within dendritic trees.

**Figure 1:**
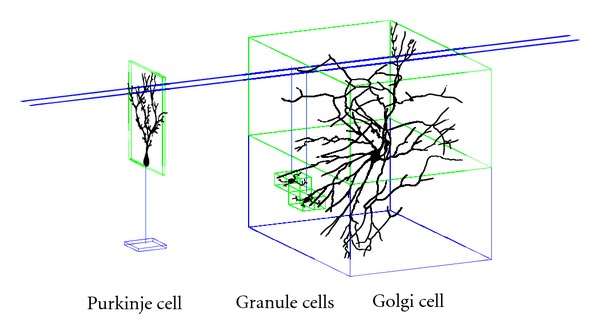
Illustration of four cerebellar neurons and their synaptic regions.

After parsing the input file, the program places all cell instances in the 3D space. The axonal and dendritic regions, where all synapses are located, are approximated as axis-aligned bounding boxes (AABBs), as seen in Figure 1. All instances of neurons of the same type have their synaptic region boxes in the same positions relative to the soma or center of the cell.

### 2.3. Visualized Examples from Cerebellar Model

Figure 1 illustrates instances of three different cell types with overlapping axonal and dendritic regions. We depict only four cells that have several overlapping regions based on their relative positions. Billions of tiny granule cells are densely packed into the lower third of the cerebellar cortex. We show just two oversized granule cells; they would be indistinguishable dots if drawn at full density and size relative to the Purkinje and Golgi cells. All pairwise overlaps of axonal (blue) and dendritic (green) regions may contain synapses if permitted by the synapse density specifications for their cell types and by the volume of the overlap between the two regions. Some overlapped regions may have no synapses, especially if the overlap volume is small; other overlaps may generate many synapses, if so defined statistically by model parameters. 

Axonal and dendritic AABBs can be broken into several smaller regions to model cell synaptic positions more accurately. In a test of a cerebellar model that contained 65 million synapses, we halved each dendritic region along *X*, *Y*, *Z* to produce an octet of eighth-sized regions that together exactly filled the same volume. We did not alter any axonal regions but did adjust synapse density parameters to produce the same number of synapses within 0.03%. The octet version of the model needed a total of 6.4 times more 1D-overlap comparisons but the total run time was only 1.23 times longer.

**Figure 2:**
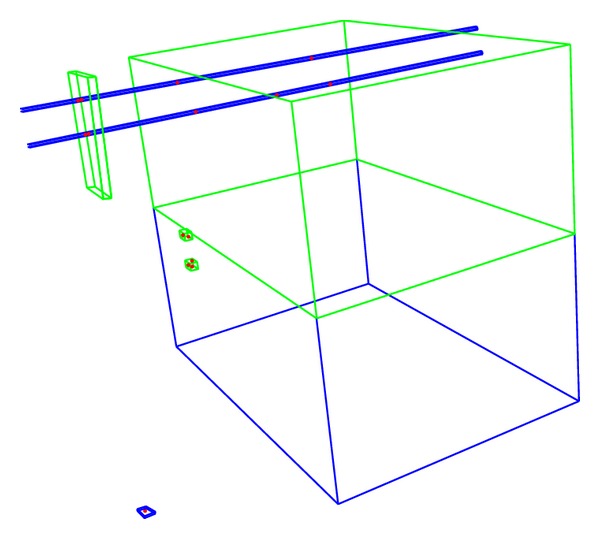
Computer-generated image of Purkinje, Golgi, and granule cell axonal (blue) and dendritic (green) synaptic regions.

We use our visualization code to inspect and analyze models created with the SWD algorithm.  Figure 2 shows an image that represents a tiny section of a cerebellar model our application created when configured to generate the model shown in Figure 1. The two horizontal lines at the top of the image are the two parallel fiber axons from the two granule cells. Each of these long axonal fibers passes through many hundreds of dendritic regions in a human brain. In the sample model of four neurons, the overlapped regions were identified, and fifteen synapses were placed in accordance with the statistical parameters provided in the input configuration file. 

## 3. Algorithms

In this section we define the problem of neurite overlap detection, discuss the limitations of previously published search methods, and explain our staggered walk methods in detail. 

### 3.1. Problem Definition

The task of finding volume overlaps in a three-dimensional space of billions of axonal and dendritic volumes can be expressed by the following generalized problem definition:

- There are billions of densely packed rectilinear boxes.
  - Sides form axis-aligned bounding boxes (AABBs)
  - Box sides are axis-aligned in three dimensions: *X*, *Y*, and *Z*
  - Boxes vary drastically in sizes and shapes.
- There are two classes of boxes: axonal (blue) and dendritic (green)
- Boxes can be colinearly located; faces may end at the same *X*, *Y*, or *Z* coordinates.
- Boxes can be enclosed within other boxes.

The goal is to identify all axonal and dendritic intersections and compute overlapping pairwise (axonal-dendritic) regions in 3D space.

### 3.2. Similar Known Algorithms

The most straightforward way to determine all pairs is to compare all axons to all dendrites. The obvious first optimization is to look for overlaps between neuronal synaptic regions only for cell type pairs that can form synapses. We refer to this pairwise constrained full comparison as the Naïve Algorithm. For each type-pair allowed to form synapses, the locations of all axonal boxes versus all dendritic boxes need to be compared to find all *XYZ* spatial overlaps that may contain synapses. The pairwise Naïve Algorithm has a cost of *O*(*AD*), where *A* is the number of axonal regions and *D* is the number of dendritic regions for the pair of cell types. When *A* ≈ *D*, the cost is *O*(*n* ^2^), where *O*( ) “order of” refers to the computational complexity and *n* is the total number of each class of region, axonal and dendritic. The complexity estimates how run time will grow as the size of a model increases.

From computer science, we have many traditional one-dimensional sweep line algorithms, such as Shamos and Hoey [20] or Bentley and Ottmann [21]. Both are impractical for our neurite-overlap detection problem, because our models may contain many densely packed overlapping 3D regions that can have colinearly located edges or can be completely nested within one another. Furthermore, our goal is not just to find all box intersections but axonal-dendritic pairs of 3D region overlaps. 

Kozloski et al. [3, 13] have devised a “touch detection” algorithm that takes a highly distributed approach to address the problem of neurite-overlap detection. In comparing their methods to ours, we will use the terms “overlap,” “3D-overlap,” and “1D-overlap.” Plain “overlap” and “3D-overlap” are equivalent; each results in an overlapping volume, or “touch.” The terms “touch” and “synapse” are roughly equivalent measures of model complexity. Our cerebellar models have an overall average of four synapses placed for every five touches. 

Kozloski's touch-detection algorithm has a similar goal to ours of locating synapses, but it targets models with much greater morphological detail and spatial precision than ours [3, 13]. Kozloski's models involve complex morphological neurons containing thousands of precisely placed small segments. Our SWD algorithm has been applied to simpler models of neurons, each described by a few dozen parameters and containing only two or three neuritic fields around each soma. Because of the higher level of detail, Kozloski et al. have created moderate-sized models that contain up to a million neurons [3]. On 4096 nodes of a Blue Gene/P, they have achieved touch detection rates of 10 billion touches per hour [3]. Our SWD algorithm has placed all synapses in a cerebellar model with 4.2 billion neurons and 3.3 trillion synaptic touches in 4.1 hours on 4096 nodes of a Blue Gene/L. With one-fourth the Blue Gene memory and processing power, SWD has already reached a touch detection rate of 680 billion touches per hour.

An important part of the approach of Kozloski et al. is the decomposition of each model into many small parts, distributing the work evenly onto thousands of Blue Gene processors. Touch-detection efficiency is improved by slicing the model into small volumes in which only segments close enough to touch are analyzed to compute precisely whether a touch occurs. To find touches rapidly in parallel, they distribute the segment data across 4096 supercomputer nodes, with four processing cores per node. 

Apart from general differences in the applicability of Kozloski's and our synapse placement algorithms, the methods differ markedly in their steps to find actual touches where a synapse should be placed. Within each volume of Kozloski's volume-based data decomposition, the initial determination of a possible touch is calculated by finding intersections between segment bounding spheres. The complexity of their algorithm is *O*(*n* ^2^), where *n* exceeds 200,000 segments per node. *O*(*n* ^2^) will become prohibitively costly for models of a billion neurons, which would need a million times more computing power than a million neurons. We believe that Kozloski's algorithm may benefit from implementing a staggered walk step in the initial touch detection phase of the algorithm.

Touch detection algorithms differ by the bounding objects being compared, for example, spheres or boxes. Many factors should be considered when choosing bounding objects that both tightly fit the model and can be compared efficiently on the available computing hardware. AABBs can be described easily by a minimum and maximum 3D point pair. Determining whether two AABBs have a 3D overlap is computationally easy because it requires only simple subtractive comparisons and allows fast rejection as soon as a comparison fails in any dimension. Spheres have an advantage that they require slightly less storage space, just a single 3D point and a 1D radius length, but their comparisons involve four multiplicative operations and have fewer fast rejection points. For instance, when comparing two AABBs, touch detection fails if the minimum *X* value for one box is greater than the maximum *X* value for the other; one failed comparison stops the computation early. 

The cerebellar cortex has a highly regular structure with specific dendritic and axonal ramification patterns and locations [7]. In our models, the regular alignment of neuritic regions allows them to be approximated by axis-aligned bounding boxes. AABBs fit our application well. An additional advantage is that the bounding boxes for the neuritic regions are not perfect cubes and mostly may be thin in one or two dimensions. Walking along a predominantly thin dimension can rapidly exclude many boxes from 1D-overlap comparisons. For example, a cerebellum has billions of long, thin axonal parallel fibers that are aligned longitudinally (*Y* axis). The dynamic version of our algorithm (SWD) checks to find the thinnest dimension. 

### 3.3. The “Staggered Walk” Algorithm

Our solution to finding axonal-dendritic 3D overlaps in which to place synapses uses a sweeping plane algorithm, similar to existing sweeping line predecessors [20, 21], but with modifications to handle densely packed overlapping boxes. During a sweep in one dimension, or a “walk,” we consider each green-to-blue and blue-to-green 1D overlap as a possible intersection and quickly reject most other box combinations that cannot overlap. The name “Staggered Walk” comes from an image of staggering along a path, checking all dendritic box signposts on one side versus axonal box signposts on the other. 

#### 3.3.1. Algorithm Steps

**Outer Loop Steps (Cell Type to Cell Type Pairs)**

1. Consider only pairs of cell types that are allowed to make connections.
2. Count the overlaps per sweep in each dimension, *X*, *Y*, and *Z*. This step is only required for SWD. For SW, choose a fixed primary, secondary, and tertiary dimension for all walks.
3. Set the order for axes to be searched in future comparisons of region face coordinates to match the axes in the ascending order of overlap counts from Step 2; the axis with the fewest 1D-overlaps along its dimension is walked first.
   Primary => Secondary => Tertiary
   (e.g., *Y* => *X* => *Z* or *Z* => *X* => *Y*)
4. Find all overlaps along the primary axis using a sweeping plane search, which we call a staggered walk*.

*The staggered walk step analysis assumes that the counts of axonal and of dendritic boxes have roughly the same value, *n*; the term *O*(*n*log⁡*n*) is a compact form of *O*(*n*log⁡(*n*)).

**Inner Loop Steps (Neuritic Region to Neuritic Region Analysis)**

- Sort the list of green boxes and the list of blue boxes by primary dimension (ordered by begin-face coordinates). *Complexity is O*(*n*log⁡*n*).
- Traverse both lists with a plane sweep in the primary dimension, queuing “open” items (ordered by end-face coordinates). *Complexity depends on the data structure used to hold open items; O*(*n*log⁡*n*)  *for a self-balanced binary search tree (BST) [22] or a skip list [23]. *
- Any new green or blue faces are compared against their complementary (blue versus green) “open” box items. *Complexity depends on the data structure; O*(*K*_*p*_)  *for BST or skip list, where K*_*p*_ *is the number of collisions in the primary dimension p. *
- The “open” items are “closed” when they are no longer able to intersect any item newly reached on the opposite side. *Complexity depends on the data structure; O*(*n*log⁡*n*)  *for BST or skip list. *
- For each overlap in the primary dimension (1D-overlap), see if there are coordinate overlaps in both the secondary and tertiary dimensions (3D overlap). *Complexity is O*(*K*_*p*_)*. *
- Calculate the volume of each overlap which occurs in all three dimensions and place the expected number of synapses randomly within the overlap volume. *Complexity is O*(*K*_*p*_).

Total cost for SW is *O*(*n*log⁡*n* + *K*_*p*_), where *K*_*p*_ is the number of collisions in the primary dimension *p*, and a self-balanced BST or a skip list is used to store “open” items.

Total cost for SWD is also *O*(*n*log⁡*n* + *K*_*p*_); optional Step 2 for the SWD variant incurs two additional *O*(*n*log⁡*n*) sorts and six *O*(*n*) traversals, but yields the same total complexity. 

Other interesting data structures that can be implemented for storage of “open” items during a staggered walk may change the costs of Steps (b), (c), and (d). At present, our C++ code uses a variable length vector for simplicity and optimal data locality. The number of vector element reorderings is small when compared to the number of traversals of the open list for comparisons (*K*_*p*_). For example, in creating a model of a 2 × 5 mm patch of human cerebellar cortex, there are over 25 billion checks for overlaps (*K*_*p*_) versus a mere 40,000 triggered vector tail reorderings, totaling a nominal 113 million items shifted. Other models may have more tail reorderings and need a self-balanced BST or a skip list for runtime efficiency.

#### 3.3.2. Primary Dimension Determination

Determining the best dimension (*X*, *Y*, or *Z*) along which first to perform the staggered walk can impact the efficiency of the algorithm depending on the model parameters. A worst-case example is where all axonal and dendritic regions overlap in the dimension chosen for the walk. In this degenerate case, our algorithm would yield a large *O*(*n* ^2^) number of comparisons, as would all other sweep algorithms previously cited. 

To minimize the danger of *O*(*n* ^2^) comparisons along the primary axis, the three dimensions can be analyzed separately, each in only *O*(*n*log⁡*n*) time, to determine which is the most efficient to use for the walk. This preprocessing is the same algorithmic complexity as the *O*(*n*log⁡*n*) sorts already required for the walk and therefore does not increase total complexity. We refer to this refinement of picking the best dimension to perform the walk per neuronal type pair as “Staggered Walk Dynamic” (SWD). 

**Figure 3:**
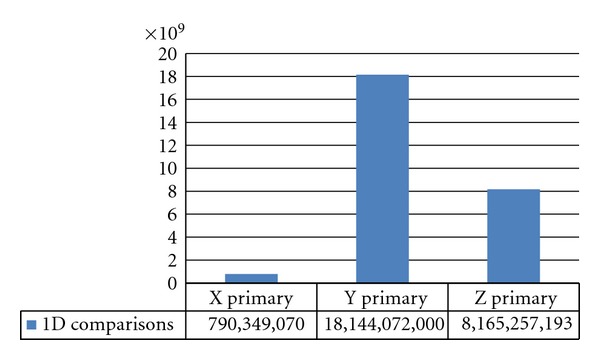
Number of primary comparisons to detect all one dimensional overlaps of granule axons with Purkinje dendrites.

Without SWD, there is no quick way to know the best possible dimension to perform the walk. Traversing all three dimensions or guessing the wrong primary dimension can require a large number of comparisons. As seen in Figure 3, when walking the dimensions to analyze granule cell axons to Purkinje dendrites, the primary dimension selected can drastically impact the number of comparisons required. Any algorithm that compares all dimensions independently incurs the maximum cost for any dimension. Linear lazy evaluation algorithms do not continue comparisons when any previous dimension has no overlap. If a lazy evaluation algorithm has a fixed comparison order (e.g., *X* then *Y* then *Z*), it will incur more overhead for some pairwise comparisons, because the optimal primary dimension often changes with the cell type pair. Highly regular regions of the brain benefit more from using SWD than those that have irregular cell orientations. If cell irregularities are localized to small spatial regions, the walk will only incur high costs within those localized regions during the primary plane sweep. 

In the SWD version, additional Step 2 determines the best dimension along which to perform the primary plane sweep for the selected cell type pair. It requires sorting both classes of neuritic boxes three times, once for each dimension; complexity is *O*(*n*log*n*) for all six sorts. A simplified staggered walk is made for each sorted list of boxes. Instead of performing edge comparisons, the simplified walk only calculates how many comparisons will be needed by adding the count of still “open” neuritic boxes on the other side for every new box that opens. The total of the counts for the simple walk measures the future cost for a full walk in its dimension. The dimension with the least sum total becomes the primary dimension. The complexity cost of each simple walk is only *O*(*n*), which is dominated by the *O*(*n*log*n*) incurred by the two sorts before each walk. For models with millions of neurons and billions of overlaps, the extra three simple walks incur only 4–6% of total SWD run times. 

#### 3.3.3. Results of the Algorithm: Cerebellar Models, Sizes, Timings, Efficiency

**Figure 4:**
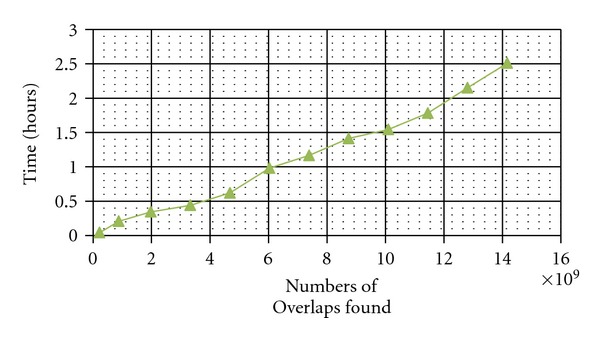
Computing hours for Staggered Walk (SWD) to find the billions of overlaps in twelve sizes of cerebellar models.

Table 1 shows that only small models can be created with the Naïve Algorithm because of its long *O*(*n* ^2^) run times. For the largest model in Table 1, taking just 150 minutes versus one month, SWD is 300 times faster than Naïve. In 4.1 hours on 4096 Blue Gene/L nodes, the SWD algorithm generated a large model for a 70 mm × 100 mm patch of cerebellar cortex with 3.3 trillion touches, resulting in 2.7 trillion synapses. On a home computer, SWD took just 2.5 hours to detect 14 billion touches for a smaller (2.8 mm × 12 mm) model. As seen in Figure 4, run times for the staggered walk dynamic algorithm scales reasonably well as model sizes increase. 

The data for Table 1 and Figure 4 were gathered from one CPU of a 3.1 GHz Windows desktop computer. The duration of the runs for the Naïve Algorithm quickly became impractically long. The monthlong time for the Naïve Algorithm to create the largest model (Table 1) was extrapolated from shorter runs. The data show that SWD can generate large models in a few minutes or hours on both a Blue Gene supercomputer and a home personal computer. 

## 4. Conclusions and Discussion

In this paper, we have targeted efficient model initialization as a key aspect of large-scale morphological neuronal modeling. We describe a staggered walk algorithm that successfully solves the problem of neurite overlap detection and synapse placement between axonal and dendritic regions in three-dimensional space. On a supercomputer, creation of models with trillions of synapses can be achieved within hours, rather than days or months.

Despite the demonstrated utility of our SWD approach, several limitations should be noted before attempting to implement this algorithm. Rather than just knowing roughly which areas project to which other areas, as is the case in simpler, faster brain modeling methods, our algorithm requires information about the geometric spans of axons and dendrites. This information may be prohibitively difficult to gather in some regions of the brain. Cerebellar cell and synapse structures are very consistent and regular; our algorithm makes use of the regularity when determining the critical primary dimension for the walk to locate synapses. Irregular axonal and dendritic regions may require many smaller bounding boxes to accurately represent their complex morphology.

The staggered walk dynamic algorithm allows us to generate dense neuronal models with trillions of overlaps and synapses in only a few hours of processing, hundreds of times more efficiently than naive *O*(*n* ^2^) searches, as shown in Table 1. With this powerful method for wiring neuronal models based on biological data, areas of the brain where there is ample information about cellular layouts are clear choices for further applications of this technology. Regions such as the neocortex, cerebellum, and hippocampus all are well studied with known topologies amenable to our method. As computational power slowly rises to levels where more detailed ultralarge-scale models are feasible, methods such as ours that combine detail and speed will be instrumental for the development of comprehensive brain simulations. 

## Figures and Tables

**Algorithm 1:**
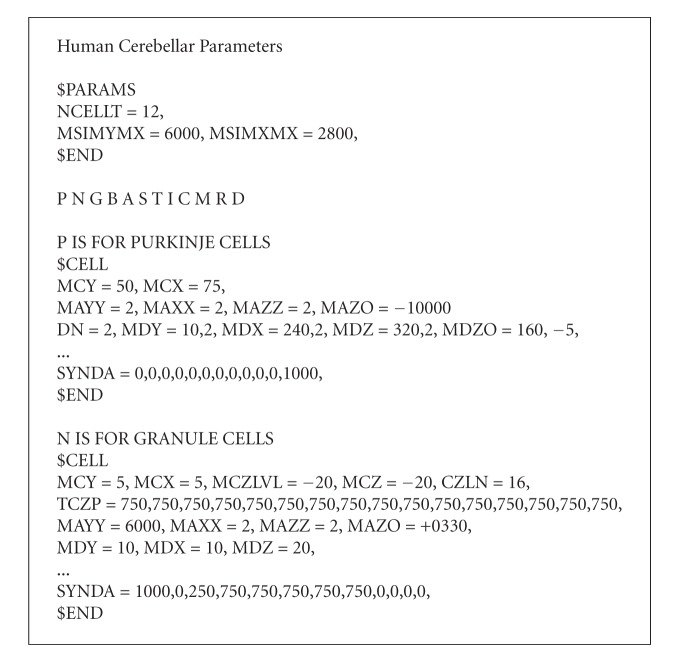
Excerpt from a parameter file for a human cerebellum model (defining only the first two cell types).

**Table 1:** Difference in performance between the naïve full comparison and our staggered walk dynamic (SWD) algorithm.

	56,462,784 Overlaps 45,675,172 Synapses (*X* = 2.8 mm, *Y* = 0.5 mm)	222,400,035 Overlaps 181,870,586 Synapses (*X* = 2.8 mm, *Y* = 1.0 mm)	496,905,207 Overlaps 405,607,980 Synapses (*X* = 2.8 mm, *Y* = 1.5 mm)	14,163,610,444 Overlaps 11,303,512,563 Synapses (*X* = 2.8 mm, *Y* = 12.0 mm)
Naïve algorithm	51 minutes	4 hours	10 hours	28–34 days*
Staggered walk dynamic	1 minute	3 minutes	6 minutes	150 minutes

*The 28–34 days value is extrapolated based on two factors: the required number of calculations of overlaps and the calculations per time demonstrated by the Naïve and SWD data runs for smaller patch sizes on the same computer.

## Appendix

### A. Detailed Description of Parameter File (Algorithm 1)

After one section ($PARAMS … $END) with overall model parameters, each input file has a section ($CELL … $END) dedicated to each cell type. The section for a cell type specifies the density and placement of its somas in the model space, the shapes and soma-relative positions of the axonal and dendritic synaptic regions for each soma, the density of synapses from its axons to each other cell type wherever their axonal and dendritic regions overlap, and electrical characteristics of its dendrites, somas, and axons.

The section for Purkinje (P) cells places the centers of all P-cell somas into a single plane at *Z* = 0 (by default), at points 50 *μ*m apart in Y (MCY) and 75 *μ*m in X (MCX). The second line (MAYY) describes each P-cell's single tiny (2 × 2 × 2 *μ*m) axonal region ending in the dentate nucleus 10,000 *μ*m (MAZO) below the P-cell center. The third line (DN = 2) gives the widths and locations of the two dendritic regions for each P-cell: the thin but huge (10 × 240 × 320 *μ*m—MDY, MDX, MDZ) main dendritic tree above (MDZO = 160 *μ*m) each soma and a tiny (2 × 2 × 2 *μ*m) synaptic region 5 *μ*m below (MDZO = −5) each soma center. P-cell electrical parameters for neurite signal propagation speeds, synapse strengths, and soma spiking behaviors are omitted (…). The last line (SYNDA) of synapse densities (in thousandths) reveals that each P-cell axon forms no (0) synapses except (1000) where it overlaps (“touches”) the dendritic region of a dentate nucleus cell (D), the last type in the model.

Parameters in the section for granule cells (N) specify that granule centers are packed 5 × 5 *μ*m apart below the P-cells in 16 (CZLN) layers 20 *μ*m (MCZ) apart from *Z* = −20 *μ*m (MCZLVL) to *Z* = −320 *μ*m. The second (TCZP = 750) line says only a randomly chosen 75% of the possible 5 × 5 × 20 *μ*m positions actually contains a granule cell. The next line (MAYY = 6000) says that each tiny granule cell has a long, thin 2 × 6000 × 2 *μ*m “parallel fiber” axon that extends 3 mm each way (+*Y*,  −*Y*), passing through the dendritic trees of nearly 400 Purkinje cells. In this model, each granule cell interacts with afferent axon endings within its 10 ×10 × 20 *μ*m dendritic synapse region. Each granule cell axon forms one synapse in all P-cell dendritic regions that it penetrates, in 25% of the Golgi cell regions, and in 75% each of the five types of inhibitory stellate interneurons found within the upper cortical layer dominated by the densely packed dendritic trees of Purkinje cells.

This sample parameter file shows an excerpt from a text file for a model of a 6,000 *μ*m by 2,800 *μ*m patch of human cerebellar cortex. The model contains somas and synapses for 12 morphologically distinct types of neurons. Each thin (2 × 2 *μ*m) granule cell axon, or “parallel fiber,” extends 6.0 mm in the longitudinal (*Y*) direction [MAYY = 6000]. Parameters are shown only for Purkinje cells plus their axonal and dendritic potential synapse regions and for the granule cells plus their synaptic regions. Electrical parameters for dendrites, somas, and axons have been elided (…). See Algorithm 1.

#### A.1. Additional Details for Figure 1

In Figure 1, the axonal (blue) and dendritic regions (green) where all synapses are located are approximated as axis-aligned bounding boxes (AABBs). All instances of neurons of the same type have their field boxes in the same positions relative to the soma or center of the cell. The two horizontal lines at the top of the image are the parallel fibers from the two granule cells. Each parallel fiber axon has a rising vertical segment before it bifurcates into horizontal segments that form hundreds of synapses with dendrites of Purkinje cells and of inhibitory interneurons up to 3 mm away longitudinally (*Y*) in both directions.

#### A.2. Additional Details for Figure 2

Axonal (blue) and dendritic (green) regions were generated from a tiny cerebellar model. Individual synapses are identified by red dots and can occur only where blue and green boxes overlap. The two horizontal lines, at the top of the image, are the horizontal segments of the parallel fibers from the granule cells. See Figure 2.

#### A.3. Additional Details for Figure 3

Three nondynamic staggered walks produced drastically different comparison counts depending on which was the fixed starting dimension (*X*, *Y*, or *Z*). The secondary dimension selected is arbitrarily based on alphabetical ordering (*XYZ* versus *XZY*; *YXZ* versus *YZX*; *ZXY* versus *ZYX*). The order of the second two axes checks has no impact on the 1D-comparison counts and resulting run times. The data were collected from a 2.8 mm by 3.0 mm model. See Figure 3.

#### A.4. Additional Details for Figure 4

Data for these models were generated on a single processor of a desktop computer using our full human cerebellar cortex parameters for different sizes of cortical patches. Data were collected for twelve models ranging from 2.8 mm × 1.0 mm to 2.8 mm × 12.0 mm. See Figure 4.

#### A.5. Additional Details for Table 1

Only models of modest sizes can be created with the *O*(*n* ^2^) Naïve Algorithm because of its long run times. Times are from a single processor of a desktop computer. The Staggered Walk Dynamic times were measured on the same computer as the Naïve times. See Table 1.
